# Reversed Frontotemporal Connectivity During Emotional Face Processing in Remitted Depression

**DOI:** 10.1016/j.biopsych.2012.04.031

**Published:** 2012-10-01

**Authors:** Nia Goulden, Shane McKie, Emma J. Thomas, Darragh Downey, Gabriella Juhasz, Stephen R. Williams, James B. Rowe, J.F. William Deakin, Ian M. Anderson, Rebecca Elliott

**Affiliations:** a Neuroscience and Psychiatry Unit, University of Manchester and Manchester Academic Health Sciences Centre, Manchester, United Kingdom; b Imaging Science, University of Manchester, Manchester, United Kingdom; c Department of Clinical Neurosciences, Cambridge University, Cambridge, United Kingdom

**Keywords:** Amygdala, connectivity, depression, emotion, fusiform gyrus, orbitofrontal cortex

## Abstract

**Background:**

Vulnerability to relapse persists after remission of an acute episode of major depressive disorder. This has been attributed to abnormal biases in the processing of emotional stimuli in limbic circuits. However, neuroimaging studies have not so far revealed consistent evidence of abnormal responses to emotional stimuli in limbic structures, such as the amygdala, in remitted depression. This suggests the problem might lie in the integrated functioning of emotion processing circuits.

**Methods:**

We recruited 22 unmedicated patients in remission from major depressive disorder (rMDD) and 21 age-matched healthy control subjects. Functional magnetic resonance imaging was performed during a face emotion processing task. Dynamic causal modeling was used with Bayesian model selection to determine the most likely brain networks and valence-specific modulation of connectivity in healthy control subjects and rMDD.

**Results:**

In healthy volunteers, sad faces modulated bi-directional connections between amygdala and orbitofrontal cortex and between fusiform gyrus and orbitofrontal cortex. Happy faces modulated unidirectional connections from fusiform gyrus to orbitofrontal cortex. In rMDD, the opposite pattern was observed, with evidence of happy faces modulating bidirectional frontotemporal connections and sad faces modulating unidirectional fusiform–orbitofrontal connections.

**Conclusions:**

Participants with rMDD have abnormal modulation of frontotemporal effective connectivity in response to happy and sad face emotions, despite normal activations within each region. Specifically, processing of mood incongruent happy information was associated with a more richly modulated frontotemporal brain network, whereas mood congruent sad information was associated with less network modulation. This supports a hypothesis of dysfunction within cortico–limbic connections in individuals vulnerable to depression.

Major depressive disorder (MDD) is characterized by impaired cognitive and emotional processing (1–4), including recognition and categorization of face emotion (5–8). Emotion categorization studies have usually but not always found reduced discrimination of face emotions in MDD (8). Some studies have additionally reported biases toward sad and/or away from happy faces. There is also evidence for altered recognition after remission from MDD (7–9), although studies are inconsistent (4,10,11). Functional magnetic resonance imaging (fMRI) has identified neuronal substrates of face processing abnormalities in MDD, particularly enhanced amygdala responses to sadness (12–14). Some evidence suggests enhanced responses to sad faces persisting into remission (14), although most studies have shown that abnormalities are state-dependent (6,11,12,15).

Although there is an established literature on abnormal function of specific brain regions during face emotion processing in MDD, there has been relatively little direct exploration of how the disorder affects connectivity between brain regions. Many neurobiological models of depression focus on network dysfunction, particularly dysfunction of a cortical–limbic “mood-regulating circuit” (16–18). In a specific model of emotion perception in MDD, Phillips *et al.* (2) argued that limbic overactivity during initial evaluation, combined with failure of cortical control, causes a bias toward processing negative information.

Recent research has investigated resting state functional connectivity in depression (19–21) and reported abnormal connectivity in regions of a mood-regulating circuit, which might relate to abnormal limbic–prefrontal white matter connectivity observed with diffusion tensor imaging (22). However, resting state studies do not optimally test network dysfunction models (2), which propose specific abnormalities during processing of emotional stimuli rather than at rest. Connectivity analyses of fMRI data can provide explicit tests of network interactions in response to emotional challenges. Functional connectivity, assessed by psychophysiological interactions (PPIs) and related methods, explores context-dependent correlations between brain regions. This technique has implicated abnormal connectivity during emotional processing in depression (23–27) and bipolar disorder (22), including abnormalities of amygdala–prefrontal connectivity in remitted patients. Cremers *et al.* (28) suggest that amygdala–prefrontal connectivity during face processing is influenced by neuroticism, a trait associated with depression vulnerability.

The PPI approaches are data-driven and give no information about direction or causality. To determine causal influences among brain regions and more directly assess theoretical anatomical models requires testing of “effective connectivity,” with models that embody causal connections such as structural equation modeling (29) or generative models such as dynamic causal modeling (DCM) (30,31). Studies using structural equation modeling have reported abnormalities in limbic–prefrontal networks in MDD during emotional face processing (32). Structural equation modeling allows a prespecified model to be tested; however, a major advantage of DCM over other connectivity approaches is the evidence-based model comparison procedure. This supports inferences of directionality and allows comparison of models to infer changes in network organization, over and above changes in connection strength within a standard network. Dynamic causal modeling employs an optimized neurovascular forward model enabling inferences to be made at the neuronal level of intrinsic and modulatory connections among multiple brain regions. A Bayesian model selection procedure allows direct comparison of different brain network models, determining which model of connectivity is most likely, given the data.

Dynamic causal modeling analyses have been carried out previously for face-processing tasks in healthy volunteers (33–36), showing emotional modulation of effective connectivity between fusiform gyrus (FG), amygdala, and orbitofrontal cortex (OFC). There have been relatively few studies using DCM in MDD, although recent studies with very simple models (typically modeling intrinsic connectivity between two nodes) have reported abnormal effective connectivity to faces in bipolar disorder and distinct abnormalities to happy faces in MDD (37). To our knowledge there have been no attempts to assess altered effective connectivity to emotional stimuli, in remitted major depressive disorder (rMDD).

The goal of the present study was to use DCM to explore abnormalities of connectivity during processing of happy and sad faces in rMDD. Rowe *et al.* (38) recently demonstrated that DCM is sufficiently robust and sensitive to study clinical populations. Indeed, a connectivity approach can be more sensitive to neuropsychiatric disorders than classical imaging analysis of regional activations (39). We adopted the Bayesian model selection approach of Rowe *et al.* (38) and predicted that happy and sad emotion would differentially modulate effective connectivity associated with face processing in rMDD compared with healthy control subjects.

## Methods and Materials

### Participants

All participants were right-handed with normal or corrected-to-normal vision and no contraindications to fMRI. Volunteers with current or past history of neurological disorder, substance dependence, or Axis 1 psychiatric disorder other than MDD or anxiety disorders were excluded, as were people taking current medications.

### Healthy Control Subjects

Twenty-nine healthy control volunteers were recruited, of whom 21 were included in DCM analysis (see Results). On the Mini-International Neuropsychiatric Interview (M.I.N.I.)-screen for Structured Clinical Interview for DSM interview (40), these volunteers had no personal or family history of psychiatric illness (Table 1).

### rMDD Subjects

Thirty remitted depressed volunteers were also recruited, of whom 22 were included in DCM analysis (see Results). These subjects met criteria for major depression in full remission on Structured Clinical Interview for DSM interview (41). For inclusion, they were required to have been remitted for at least 3 months, with current scores of <12 on the Montgomery-Åsberg Depression Rating Scale (MADRS) and to be medication-free (Table 1). A *t* test revealed that, although within the normal range, rMDD volunteers had significantly higher MADRS scores than healthy control subjects (*t* = −2.15, *p* = .04). A history of anxiety disorder was not an exclusion criterion; however, only 4 of 22 subjects in the DCM analysis had history of clinically significant anxiety, and none had current symptoms.

### Emotional Face Processing Task

A series of faces was presented, each displayed for 3 sec with a 500-msec gap. There were four different emotion conditions—neutral (N), happy (H), sad (S), and fear (F)—and a rest condition (R), which was fixation on a central cross. The emotions of interest in this study were happy and sad, because abnormalities in processing these mood-relevant emotions have been most consistently associated with MDD. Each face block lasted 21 sec and consisted of six faces (three male; three female). Standard face stimuli of Ekman and Friesen were used, in conjunction with a morphing procedure (42) such that faces displayed emotions at 80% intensity. There were 22 blocks presented in an NHNSNFNRSNHNFNRFNSNHNR order; total duration was 7 min 42 sec. Volunteers responded with a button press to each face indicating whether it was male or female. We present results for sad and happy emotions, in line with fMRI literature emphasizing the particular significance of these mood-related emotions to depressive disorders.

### Imaging

Images were acquired on a 1.5T Philips Intera scanner (Eindhoven, the Netherlands) with a single-shot echo-planar sequence. Two hundred eighteen volumes were acquired, each comprising 29 contiguous axial slices (repetition time/echo time = 2100/40 msec, 3.5 × 3.5 mm in-plane resolution, slice thickness 4.5 mm, .5 mm slice gap). A T1-weighted structural scan (256 × 256 matrix, repetition time = 8.99 msec, echo time = 4.2 msec, 160 axial slices, voxel size .875 × .875 × 1 mm) was acquired for each participant to be used in spatial normalization.

### Analysis of Region-Specific Responses

Pre-processing was carried out with SPM8 (http://www.fil.ion.ucl.ac.uk/spm). All images were realigned to the first to correct for movement, spatially normalized to standard space (Montreal Neurological Institute) and smoothed with a Gaussian kernel of full width at half maximum 7 × 7 × 10 mm. A high-pass filter of twice the maximum stimulus repetition time was applied.

To determine coordinates of regional maxima for DCM analysis, random effects analysis was carried out to identify regional changes in blood oxygen level–dependent response to all faces compared with rest across the whole subject group. We performed a region of interest (ROI) analysis in four regions comprising a network of face-processing regions where effective connectivity is modulated by emotional valence (34,36): primary visual cortex (V1), FG, amygdala, and OFC. Maxima were identified in each ROI in the random effects analysis. For each individual, local maxima within 14 mm of the maximum group activation were found, and data were extracted from a 6-mm radius sphere centered at these local maxima. Regional responses at these maxima are reported in Table 2.

### DCM of Effective Connectivity

Dynamic causal modeling was used to examine the effect of rMDD on effective connectivity with a set of models specified in SPM8, following methods recommended by Stephan *et al.* (30,43), which have reliably detected between-group differences in connectivity (38). The analysis schema is given in Supplement 1.

The anatomical network model proposed by Fairhall and Ishai (34) includes V1, superior temporal sulcus, FG, amygdala, inferior frontal gyrus, and OFC. However, connections between V1, FG, amygdala, and OFC were particularly sensitive to modulation by emotion, and therefore we specifically include only these areas; see also Dima *et al.* (36). Left hemisphere responses were less extensive and less consistent across subjects, as has previously been observed (44,45). Reliable DCM analysis requires exclusion of subjects who do not show response in all network foci, and we therefore performed DCM only on the right hemisphere network. We have previously demonstrated with the model used here that adequate power can be achieved to detect subtle group differences (46).

**Figure 1:**
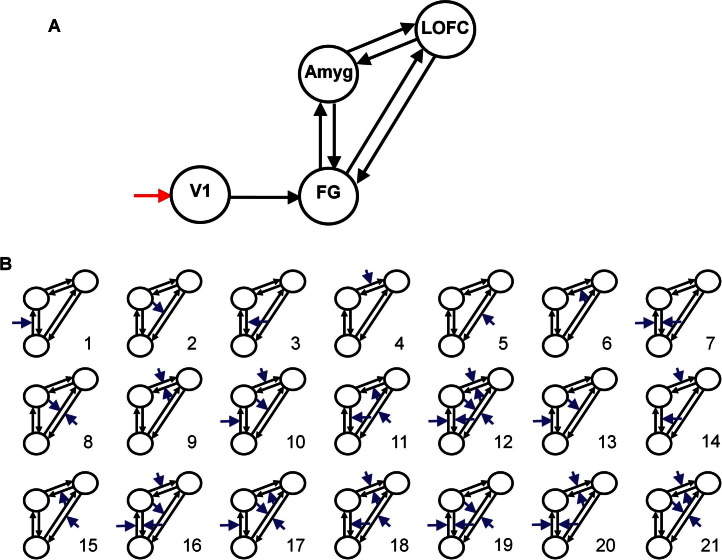
The models tested with Bayesian model selection. **(A)** Fully connected model of intrinsic connectivity with feedforward and feedback connections for each part of the network; **(B)** the patterns of modulatory influences of emotion on connectivity among fusiform gyrus (FG), amygdala (Amyg), and lateral orbitofrontal cortex (LOFC). These 21 models belong to one of seven model “families” (see Methods). V1, visual cortex.

A three-stage approach to model selection was used. First, connections between the ROIs were specified as intrinsic connections with all faces compared with rest modeled as input to V1. We tested seven structurally distinct models (Supplement 1). Bayesian Model Selection within each group clearly identified the fully interconnected model as most likely, with feedforward and feedback connections (Figure 1A).

In the second step, the fully connected anatomical model, with intrinsic connectivity as in the preceding text, was used to make 21 models. Each of these models was subject to distinct profiles of modulation by emotion (Figure 1B). The 21 models were fitted with data from each subject, generating the model log-evidences and posterior probabilities—parameters that provide an index of the accuracy of the model, adjusting for model complexity and dependencies among parameters.

The models were then partitioned into different families, according to shared model features. This facilitates optimum model selection and interpretation. Details are given in Supplement 1. Having identified the optimum model family, we then compared individual models within those families, again with Bayesian model selection (30,38,43,47).

### Functional Connectivity Analysis with PPI

The DCM with Bayesian model selection approach allows us to test our hypotheses that the best-fitting models for happy and sad faces differ between patients and control subjects but rests on several assumptions and limitations in model design and fitting. Following the approach of Passamonti *et al.* (48,49), DCM analysis was therefore supplemented by an analysis of PPIs with general linear models, with fewer anatomical constraints and no inverse modeling of neurovascular coupling but without model comparison or inferences of directionality. Separate PPIs were carried out for happy versus neutral and sad versus neutral with the right amygdala as a seed region. Data from the amygdala were extracted from an 8-mm sphere, constructed around the focus used in the DCM analysis (25, −4, −15). A time series was calculated for each participant with the first eigenvariate from the time series of all voxels within the sphere. The PPI regressor was calculated as the product of the right amygdala neuronal time series and a vector coding for the task comparison (happy–neutral or sad–neutral). Subject-specific PPI contrast images were entered into second-level analysis, identifying brain areas where the change in emotion-related connectivity with the amygdala differed between groups. We also examined whether in the rMDD group, there were correlations between connectivity and MADRS depression ratings. This was a post hoc ROI analysis to supplement the DCM findings, and we therefore used a composite ROI comprising FG and OFC, defined anatomically, thresholded at *p* < *.*05 corrected.

## Results

### fMRI Activations

When responses to emotional faces were contrasted with rest in all subjects, right-sided maxima were observed in the pre-specified ROIs: V1 (18, **−**95, 0), FG (42, **−**60, **−**15), amygdala (25, **−**4, **−**15), and OFC (28, 32, **−**15) (Table 2) (note: left hemisphere maxima also reported for completeness). No other regions of significant activation were observed, and there were no differences between the groups at *p* < *.*001 uncorrected.

For DCM analysis, we selected subjects who exhibited activity (*p* < *.*05 uncorrected) within 14 mm of all regional maxima in the t contrast of emotional faces compared with rest. For the right hemisphere, significant response within 14 mm of all maxima was observed in 21 of 29 healthy control subjects and 22 of 30 rMDD volunteers, and only these participants were included in DCM. Results of the standard subtraction analysis in the full sample are reported and discussed elsewhere (11).

### DCM Analysis

**Figure 2:**
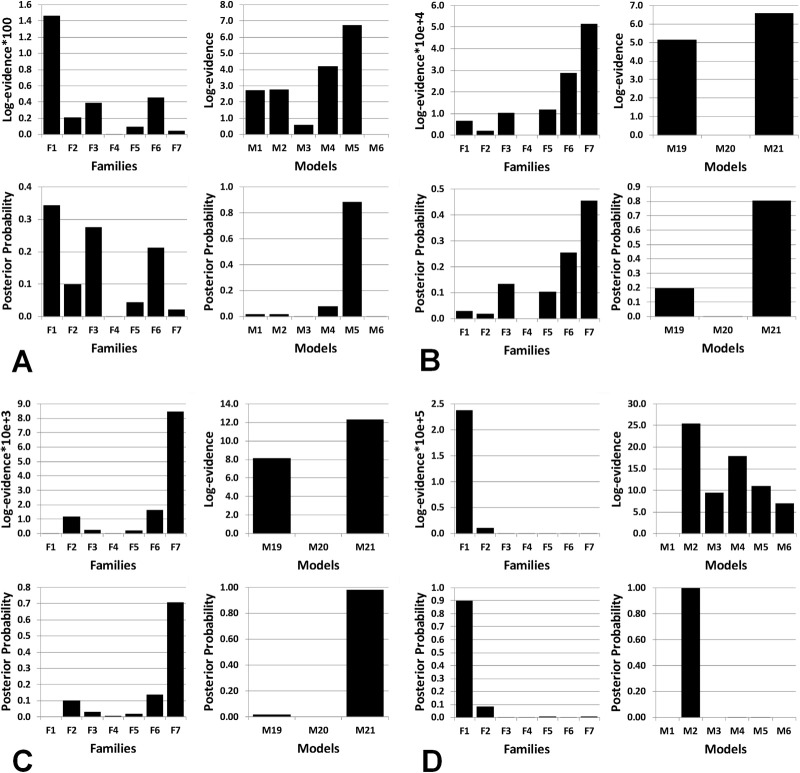
Results of Bayesian Model Selection for identification of the most likely family (F1–F7) and, within the winning family, the most likely network models (Models 1–3 in Family F7, Models 1–6 in Family F1). Results are shown separately for: **(A)** Healthy Control Group Happy Condition; families and models; **(B)** Healthy Control Group Sad Condition; families and models; **(C)** Remitted Depressed Group Happy Condition; families and models; and **(D)** Remitted Depressed Group Sad Condition; families and models. For each of **A–D**, the differences in log-evidences between the first- and second-place models within Families 1 and 7 are highly significant (very strong evidence for a difference), with standard Bayesian thresholds (50), as can most easily be seen from the differences in the posterior probabilities of each family/model.

Table 3 and Figure 2 show the results of Bayesian model selection for each group, indicating very significant differences in model evidences (50), with opposite directions of effect in the two groups.

For healthy control subjects viewing happy faces, the most likely family was Family 1, and within that family, the most likely model was Model 5 (posterior probability .86). This network is defined by modulation of the single backward connection from OFC to FG. For healthy control subjects viewing sad faces, the most likely family was Family 7, and within that family, the most likely model was Model 21 (posterior probability .81). This network is defined by modulation of bidirectional connections between OFC and amygdala and between OFC and FG.

For rMDD subjects viewing happy faces, the most likely family was Family 7, and within that family, the most likely model was Model 21 (posterior probability .98). Thus the most likely model for rMDD participants viewing happy faces was the same model that best fitted control subjects viewing sad faces. For rMDD subjects viewing sad faces, the most likely family was Family 1, and within that family, the most likely model was Model 2 (posterior probability 1.0), with modulation of the single forward connection from the OFC to FG. Thus the best-fitting model for rMDD participants viewing sad faces was similar to the model that best fitted control subjects viewing happy faces, with only the unidirectional OFC–FG connection modulated but in a different direction.

### PPI Results

**Figure 3:**
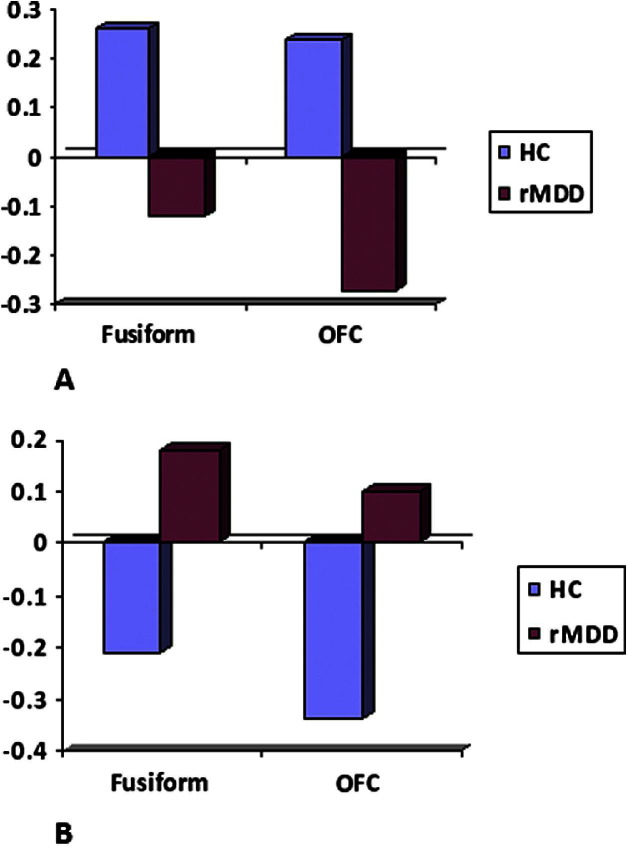
Results of psychophysiological interaction analysis. **(A)** Relative strengths of connectivity from the amygdala for processing sad versus neutral faces in remitted major depressive disorder (rMDD) and healthy control subjects (HC). **(B)** Relative amygdala connection strengths for processing happy versus neutral faces. OFC, orbitofrontal cortex.

For happy faces, right amygdala connectivity to FG (31, −56, 5) and OFC (25, 32, −10) was significantly reduced (*p* < *.*05, family-wise error corrected) in healthy control subjects compared with rMDD. For sad faces, right amygdala connectivity to FG (28, −63, −15) and OFC (11, 40, −5) was significantly reduced (*p* < *.*05, family-wise error corrected) in rMDD patients compared with healthy control subjects (Figure 3). There were no correlations between amygdala connectivity and either Clinical Anxiety Scale or MADRS scores in any brain regions even at a liberal threshold of *p* < *.*01 uncorrected.

## Discussion

In this study we identified how emotional valence modulated connectivity within a network of regions involved in face processing in healthy control subjects and rMDD. Happy and sad emotions modulated connectivity differently in the two groups, even though there were no significant differences in either region-specific responses or intrinsic connectivity associated with face processing.

Inferences about the directionality of connections (forward, backward, bidirectional, or absent) are important in the context of emotion networks and made possible by DCM model comparison procedures. Because we were primarily interested in the relationship between rMDD and the emotional modulation of network connectivity, we used a first stage of DCM modeling to identify the most likely network. Within this fully connected network, we then characterized the pattern of emotional modulation of connections in each group. Strikingly, the family-based Bayesian model selection suggested a reversal of the association between emotion, connectivity, and group. Although DCM does not directly compare model fits from different datasets (e.g., different groups), the reversal of the Bayesian model comparisons between groups implies highly significant differences in underlying neural architectures for emotional processing in the two groups (48). We went on to corroborate our findings with PPIs, a complementary technique that does not support inferences of causality or directionality but uses simpler general linear models to infer differences in functional connectivity.

Dynamic causal modeling indicated that the pattern of modulation by sad faces in control subjects was the same as for happy faces in patients, involving bi-directional connections between OFC and both amygdala and FG. By contrast, happy faces in control subjects and sad faces in rMDD participants were associated with modulation of unidirectional connection between the OFC and FG (the backward connection in control subjects and the forward connection in rMDD). This was corroborated by a direct comparison of functional connectivity between the two groups with PPI. This analysis showed relatively reduced amygdala connectivity in rMDD during processing of sad faces but relatively enhanced amygdala connectivity during processing of happy faces.

Our finding that emotional valence specifically modulates effective connectivity in a face processing network in healthy control subjects is broadly consistent with previous reports (33–35). However, there are some discrepancies. Fairhall and Ishai (34) reported enhanced forward connectivity between inferior occipital gyrus, FG, and amygdala, whereas we observed enhanced OFC–FG feedback connectivity for happy faces and enhanced bidirectional connectivity to OFC to both FG and amygdala for sad faces. One reason for the difference is that Fairhall and Ishai (34) combined positive and negative emotions, simply assessing effects of “emotional” faces. Here we show that happy and sad emotions modulate effective connectivity in different ways, suggesting that—as for standard fMRI analysis—combining different emotions might obscure significant effects. Dima *et al.* (36) recently showed that different negative emotions modulate connectivity in specific ways, with sad faces particularly modulating the FG–OFC connection. A second critical difference is in the nature of the task demands. The Fairhall and Ishai paradigm was passive viewing. Here, as in Dima *et al.* (36), we required subjects to make active decisions about faces. Evidence suggests that precise cognitive and attentional demands of a task might be critical determinants of neuronal response to emotional faces, both in region-specific fMRI analysis (35) and effective connectivity modeling (51).

Our main finding is that emotion-specific modulation of effective connectivity is abnormal in rMDD. Neither behavioral performance nor region-specific neuronal responses to emotional faces have conclusively distinguished rMDD from control subjects ([4] for review). A number of studies, including our own, suggest that rMDD subjects show increased recognition of emotions, particularly negative emotions (8,9,52), although the effects are subtle. Studies of region-specific neural abnormalities have reported mixed results, with both enhanced (14) and normal (8) amygdala responses observed in rMDD. Probably the most consistent finding is normalization of abnormalities found in current MDD with short-term successful treatment (6,12,13). In the cohort described here, there were no significant group differences in region-specific responses to happy or sad faces.

However, in the absence of region-specific differences, the best fitting model families for the two groups are reversed for the two emotions, providing evidence for group differences in connectivity. In support of the DCM results, a PPI analysis reveals a reversal of emotion-specific face processing-related amygdala connectivity between the two groups on direct statistical comparison. Behaviorally, healthy control subjects are biased toward processing happy information in various contexts, whereas rMDD might be associated with bias toward negative, specifically sad, information (4). If we consider—as a shorthand—happy and sad faces as “congruent” with the biases seen in control subjects and rMDD subjects, respectively, and the other emotion “incongruent,” our results can be interpreted as indicating that the processing of congruent emotion in both control subjects and rMDD subjects is associated with modulation of FG–OFC connectivity only. Conversely, incongruent face emotion is associated with the same connectivity modulation in both groups: increased modulation of bidirectional connectivity of the OFC to both FG and amygdala. Thus, for control subjects and rMDD, only incongruent emotion modulates the amygdala–OFC connection. Enhanced processing of mood-congruent information might depend on decreased inhibitory cortico–limbic connectivity in both patients and control subjects. Almeida *et al.* (53) have previously reported abnormal top-down OFC–amygdala connectivity during overt processing of happy faces in MDD, which they interpret as reflecting increased inhibition of positive emotion. This is consistent with our suggestion that mood-incongruent information might be associated with greater inhibitory connectivity, whereas mood-congruent information is associated with decreased inhibitory connectivity. It cannot be concluded from the present results whether different patterns of connectivity between patients and control subjects represent a general mechanism for mediating subtle attentional biases in rMDD or whether the findings are specific to face emotion-processing. However, the results indicate clearly that connectivity approaches (both DCM and PPI) might importantly detect abnormalities that are not observed with “classic” region-specific fMRI.

Abnormal connectivity between networks connecting OFC, FG, and amygdala are consistent with previous studies using various techniques. Anatomical connectivity can be assessed with tractography techniques such as diffusion tensor imaging. Gschwind *et al.* (54) demonstrated white matter (structural) connectivity between visual processing areas and amygdala, although FG–amygdala connectivity was weak (perhaps consistent with the absence of modulation of this connection in the present study). The OFC–amygdala connection is well-established (55) and has been demonstrated in a recent tractography study (56). Diffusion tensor imaging studies in depression suggest abnormalities in structural connectivity of OFC–amygdala, both in currently depressed patients and those at high risk (22,57). Direct connections between FG and OFC are less well-established; however, both regions connect via major fiber tracts to temporal pole (58), and therefore indirect anatomical connection is plausible.

Studies of functional and effective connectivity also support a hypothesis of abnormal connectivity within the network suggested here. Resting state connectivity studies (19–21) describe aberrant connectivity associated with depression. More relevant studies of functional and effective connectivity in response to emotional challenges also suggest impaired fronto–limbic connectivity (23–27,32). An important advantage of our study is that we did not rely on a between-groups comparison of parameter estimates of connection strengths. Early applications of DCM for group effects have used the connectivity parameter as a dependent variable, with a single connection (53) or the same model for patients and control subjects (59). However, realistic models can be more complex, with multiple connections and modulatory influences. In these circumstances, reliability of parameter estimates can be poor (38), and model-level inference, such as our staged Bayesian model selection procedure, is more appropriate (30,43).

A recent study, with a similar approach to ours, showed that modulation of normal serotonin (5-HT) function by acute tryptophan depletion altered the connectivity of amygdala and ventrolateral frontal cortex during processing of emotional faces (48). Regions of the network tested here are rich in 5-HT receptors (60), and studies (61–65) show that manipulation of 5-HT in healthy volunteers modulates responses to emotional faces in these regions. Fisher *et al.* (66) used multimodal imaging to show that 5-HT receptors are specifically implicated in emotion-modulated coupling of amygdala and OFC. It therefore seems plausible that the differences in connectivity we observe depend on 5-HT mechanisms. There is abundant evidence for impaired 5-HT neurotransmission in depression (4), although evidence for ongoing abnormalities of 5-HT function in rMDD is more mixed. Our present results might suggest ongoing subtle abnormalities of 5-HT mediated connectivity in response to emotional stimuli, although this hypothesis requires explicit testing.

One limitation of this study is that not all participants activated all regions in response to faces at a standard threshold. The requirements of plausible DCM analysis necessitate exclusion of those who do not activate all nodes, but this raises questions about whether included participants are a distinct subset of rMDD patients. It should be noted that: 1) similar proportions of rMDD and healthy control subjects were excluded, and 2) included participants did not differ from the whole group on any demographic measures. A further limitation is that, although scoring within the normal range, rMDD patients had significantly higher MADRS scores than control subjects. In a traditional fMRI analysis, it would be possible to examine the contribution of residual symptomatology with correlations, but this cannot yet be applied within DCM model selection. However, in our PPI analysis, covarying for symptom scores did not influence the principal results.

In conclusion, we have shown that rMDD is associated with a reversal of the normal pattern of valence-specific modulation of face-processing connectivity. The rMDD participants show greater bidirectional OFC–amygdala connectivity for happy faces than control subjects and less OFC–amygdala connectivity for sad faces. This pattern might reflect increased inhibitory control of mood-incongruent information and decreased inhibitory control of mood-congruent information. Our findings further support the use of DCM to explore aberrant connectivity in depression. Future studies are required to determine whether abnormal connectivity predicts relapse to MDD or severity of depressive episodes.

## Figures and Tables

**Table 1:** Participant Characteristics

	Healthy Control (*n* = 21)	rMDD (*n* = 22)
Male:Female	7:14	6:16
Mean Age (SEM)	31.1 (9.97)	33.73 (10.69)
MADRS (SEM)	.92 (1.44)	2.31 (3.24)
Past Episodes	—	3.13 (2.6)

MADRS, Montgomery-Åsberg Depression Rating Scale; rMDD, remitted major depressive disorder.

**Table 2:** Coordinates of Activations Found for DCM Analysis

Region	BA	x	y	z	Cluster Size (Voxels)	*t* Score
Left V1	17	−14	−98	0	865	12.95
Right V1	17	18	−95	0	865	11.45
Left Fusiform		−35	−77	−20	865	7.64
Right Fusiform		42	−60	−15	865	8.02
Left Amygdala		−18	−7	−15	142	4.03
Right Amygdala		25	−4	−15	113	4.67
Left OFC	47	−42	21	−15	33	1.97
Right OFC	47	28	32	−15	46	2.06

Threshold set at *p* < *.*05 uncorrected. BA, Brodmann area; DCM, dynamic causal modeling; OFC, orbitofrontal cortex; V1, visual cortex.

**Table 3:** Bayesian Model Selection within Best-Fitting Families of Models

	Healthy Control			Remitted Depressed		
	Most Likely Family	Most Likely Model	Posterior Probability	Most Likely Family	Most Likely Model	Posterior Probability
Happy	1	5	.86	7	21	.98
Sad	7	21	.81	1	2	1
