# Supplementary material for: Reversed Frontotemporal Connectivity During Emotional Face Processing in Remitted Depression
Source: Biol Psychiatry. 2012 Oct 1;72(7):604–11. doi: 10.1016/j.biopsych.2012.04.031 (PMC3657140; doi:10.1016/j.biopsych.2012.04.031)
Supplement: Supplement 1 [file mmc1.pdf]

Reversed Frontotemporal Connectivity During Emotional Face Processing in Remitted Depression

Supplemental Information

Supplemental Methods

Schema for Dynamic Causal Modelling Analysis (as proposed by Stephan *et al*. (1, 2))

1. Define an anatomical network of contributory regions.

2. Extract blood oxygen level-dependent functional magnetic resonance imaging time series for each subject in each network region.

3. Define a set of models of variations in intrinsic connectivity of this network.

4. Specify bilinear terms showing modulatory effects of valence (happy, sad).

5. Specify driving inputs to the network, in our case inputs to primary visual cortex corresponding to face stimuli.

6. Estimate the models' parameters (including the forward neurovascular model and the intrinsic, driving and modulatory connections) and the negative free energy estimate of the log-model evidence, adjusting for model complexity.

7. Compare models within each group (remitted major depressive disorder and control) using the free energy estimate of the model evidence.

Intrinsic Connectivity

We tested seven structurally distinct models (Figure S1) and used Bayesian model selection to determine the most likely model for each subject group. Results of this analysis are presented below (Figure S2). For both groups, the fully interconnected model with feedforward

and feedback connections (Figure S1 7) was by far the most likely model given the data (exceedance probability > 0.9, Figure S2).

Modulatory Connectivity: Specification of Model Families

Prior to determining which of our 21 models of modulatory connections best fit the data for each emotion and each group, we divided the models into families. In a family of models, the individual models share some critical feature that distinguishes them from other models (3). This approach to identifying the optimal model was chosen above a 147-way comparison across all models, as a structured, systematic and tractable approach to both the large model-space and complex model comparison. The model families used here were:

1. Single connection modulated: models 1-6
2. Models with modulation of reciprocal connections: models 7-9
3. Models with all forward or all backward connections modulated: models 10-11
4. All connections modulated: model 12
5. Modulation of connections from one region: models 13-15
6. Modulation of connections from two regions: models 16-18
7. Modulation of 2 sets of reciprocal connections: models 19-21

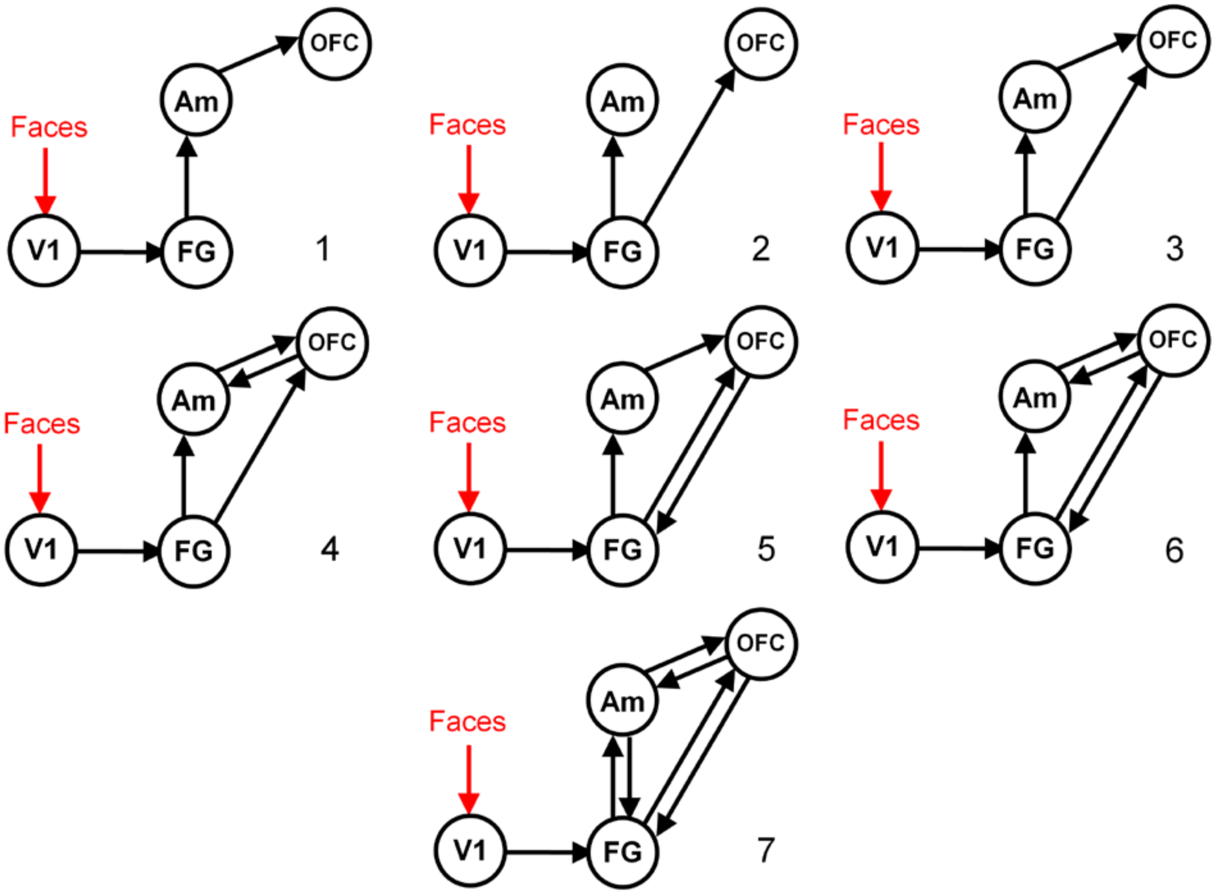

Figure S1. The 7 models of intrinsic connectivity tested. Am, amygdala; FG, fusiform gyrus; OFC, orbitofrontal cortex; V1, primary visual cortex.

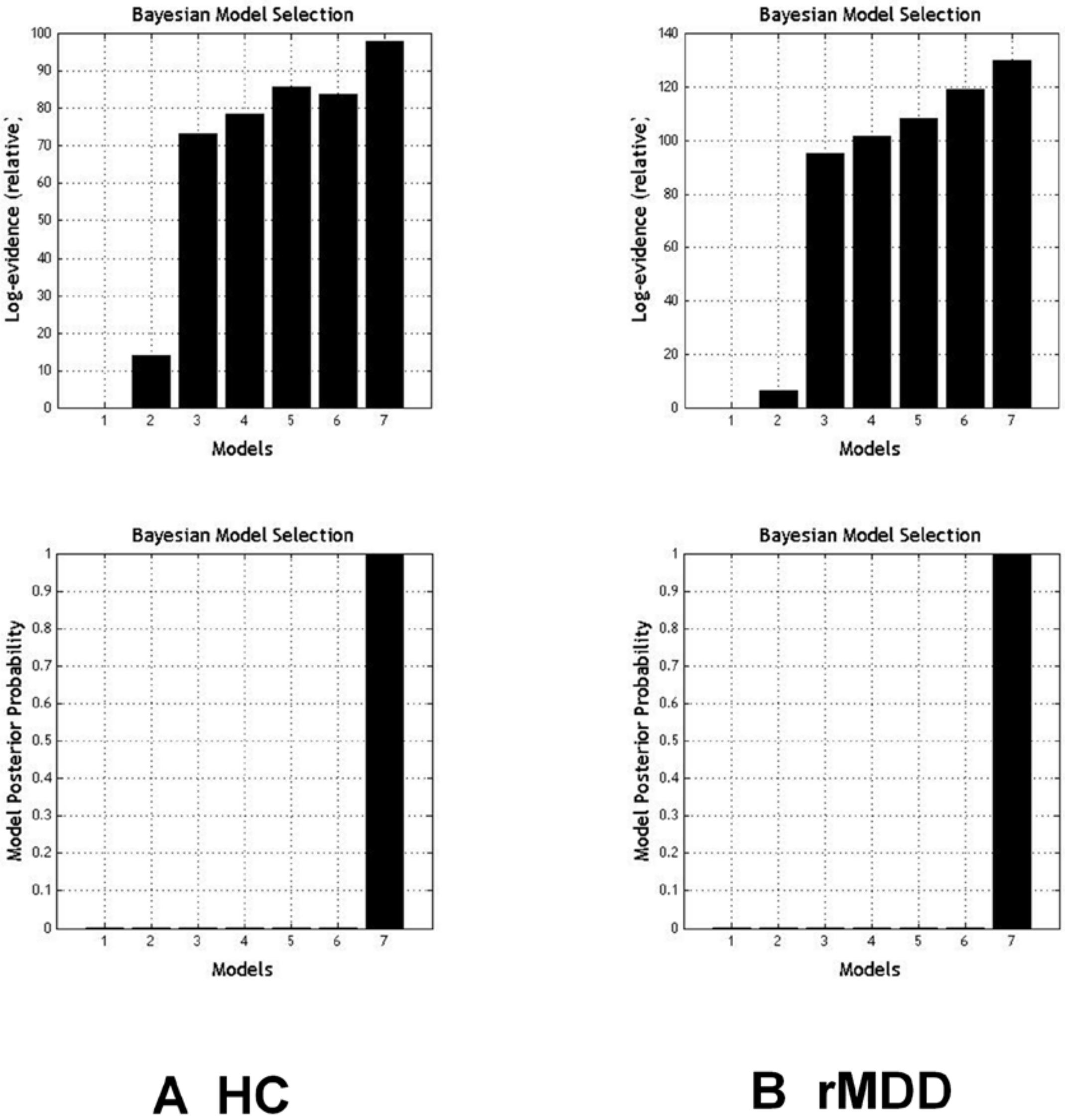

Figure S2. Results of Bayesian model selection for intrinsic connectivity models, showing that model 7 (fully connected) was the most likely model for each group, as evidenced by posterior probability approaching 1. HC, healthy controls; rMDD, remitted major depressive disorder.

Supplemental References

1. Stephan KE, Penny WD, Moran RJ, den Ouden HE, Daunizeau J, Friston KJ (2010): Ten simple rules for dynamic causal modeling. *Neuroimage* 49:3099-109.
2. Stephan KE, Penny WD, Daunizeau J, Moran RJ, Friston KJ (2009): Bayesian model selection for group studies. *Neuroimage* 46:1004-17.
3. Penny WD, Stephan KE, Daunizeau J, Rosa MJ, Friston KJ, Schofield TM, Leff AP (2010): Comparing families of dynamic causal models. *PLoS Comput Biol* 6:e1000709.
